# Early Life Risk Factors in Pediatric EoE: Could We Prevent This Modern Disease?

**DOI:** 10.3389/fped.2020.00263

**Published:** 2020-05-29

**Authors:** Martina Votto, Gian Luigi Marseglia, Maria De Filippo, Ilaria Brambilla, Silvia Maria Elena Caimmi, Amelia Licari

**Affiliations:** Department of Pediatrics, Fondazione IRCCS Policlinico San Matteo, University of Pavia, Pavia, Italy

**Keywords:** eosinophilic esophagitis, allergy, risk factors, early life exposures, food allergens, microbiome, prevention

## Abstract

Eosinophilic esophagitis (EoE) is a chronic antigen-mediated inflammatory disease that affects the esophagus. In the last 20 years, a large number of epidemiological studies showed a significant increase in the incidence and prevalence of EoE, especially in developed countries. This phenomenon might correlate to the overall increase in pediatric allergic diseases or might be a result of improved medical awareness and knowledge through modern diagnostic instruments. Since 1993, when EoE was first recognized as a distinct clinical entity, several signs of progress in the pathophysiology of EoE were achieved. However, a few studies reported data on early risk factors for pediatric EoE and how these factors may interfere with genes. Currently, the most defined risk factors for EoE are male sex, Caucasian race, and atopic comorbidities. Other putative risk factors may include alterations in epithelial barrier function and fibrous remodeling, esophageal dysbiosis, variation in the nature and timing of oral antigen exposure, and early prescription of proton pump inhibitors and antibiotics. Notably, the timing and nature of food antigen exposure may be fundamental in inducing or reversing immune tolerance, but no studies are reported. This review summarized the current evidence on the risk factors that might contribute to the increasing development of EoE, focusing on the possible preventive role of early interventions.

## Introduction

EoE is a chronic, antigen-mediated, inflammatory disease of the esophagus characterized by symptoms due to esophageal inflammation, dysmotility, and fibrosis (1, 2). EoE occurs in children and adults, and symptoms are often non-specific and depending on the age of onset (1, 2). While in toddlers and children EoE presents with inflammatory symptoms mimicking gastroesophageal reflux disease (GERD), in adolescents and adults EoE frequently appears with food impaction, dysphagia, odynophagia, or esophageal strictures, as a consequence of the ongoing fibrosis process (1, 2). EoE is a multifactorial disorder resulting from the combination of genetic predisposition, epithelial barrier dysfunction, environmental risk factors (Table 1), and allergen sensitization, leading to a T helper type 2 (Th2) atopic inflammation of the esophagus (2).

**Table 1:** Risk factors of eosinophilic esophagitis [adapted from Dellon and Hirano (3)].

Male sex	Gene encoding for thymic stromal lymphopoietin (TLSP), a central mediator of eosinophilic inflammation, is located on a pseudo-autosomal region of the X and Y chromosomes (Xp22.3 and Yp 11.3). A single nucleotide polymorphism of this region predisposes male patients to develop EoE (4)
Family members of patients with EoE	Monozygotic twins had a 44% disease concordance, a 2-fold increase compared with dizygotic twins (5, 6). Also, the relative risk to develop this disease in dizygotic twins might increase more than 10-fold compared to siblings (5)
Genetic *loci*	Studies of Genome-wide association studies (GWAS) identified different genetic *loci* that are likely contributing to the development of EoE and mainly include thymic stromal lymphopoietin (TSLP), calpain 14 (CAPN14), EMSY, LRRC32, STAT6 and ANKRD27 (7). These genetic loci are mainly involved in T-helper 2 type inflammation (allergic inflammation) and epithelial barrier function and integrity
Non-atopic diseases	EoE prevails in patients with connective tissue disorders, coeliac disease, autoimmune diseases, autism, and ADHD (8)
Atopic diseases	EoE may be a late manifestation of the atopic march (9)
OIT for foods and aeroallergens	EoE is a complication of oral immunotherapy (OIT) in 3–5% of cases. EoE is also reported during sublingual immunotherapy (SLIT) for respiratory allergies (10)
Infectious Esophagitis (HSV)	HSV might impair the esophageal barrier and increase the epithelial permeability (11, 12)
GERD	GERD alters the esophageal barrier function, increases the epithelial permeability, and the passage of food allergens that might trigger EoE. Furthermore, GERD might induce the expression of inflammatory molecules and eosinophil chemoattractants (13–15)
Aeroallergens	Environment allergens might increase disease activity and explain the seasonal variation of EoE reactivations and diagnosis (16, 17)
Food allergens	Food allergens directly trigger EoE (1)
Cold climate regions	Higher exposition to aeroallergens (18)

Since 1993, when EoE was first recognized as a distinct clinical entity, several signs of progress in the pathophysiology of EoE were achieved; however, few studies reported data on early risk factors and how these factors might interfere with the genes in the disease onset and evolution. EoE is strictly associated with atopic disorders (asthma, atopic dermatitis, IgE mediated food allergy, allergic rhinitis), suggesting that EoE and allergic diseases share the same environmental risk factors and early life exposures.

We reviewed the recent evidence about the well-known risk factors of EoE, also reporting the less-investigated early exposures, to open future ideas of investigation in the limited field of prevention. Finally, we speculate about the possible strategies for EoE prevention.

## Why is EoE a Modern Disease of Western Countries?

Recently, it was estimated that EoE affects 1/2,000 patients in the United States, with higher prevalence rate in adults (43.4/100,000; 95% CI, 22.5–71.2) than in children (29.5/100,000; 95% CI, 17.5–44.7), prevailing in Caucasian patients and male sex (Table 1) (1, 3, 19). In the last 20 years, a large number of epidemiological studies showed a significant increase of incidence and prevalence of EoE especially in children in Western Countries, varying widely across North America and Europe (19–21). This interesting phenomenon might be related to (1) an overall increased incidence of allergic and non-allergic diseases, (2) the chronic disease-course of EoE, and (3) the improved medical awareness and knowledge through modern diagnostic instruments (18). Although EoE is associated with some genetic polymorphisms (22, 23), this rapid increase in EoE frequency might indicate a prevalent role of environmental risk factors in disease development.

### Hygienic Hypothesis, Dysbiosis, and Esophageal Infection

The hygienic hypothesis postulated for the first time in 1989 by Strachan (24), and recently reviewed (25), has explained the global rise of allergic and autoimmune diseases. Animal and human studies demonstrated that the increased frequency of allergic diseases in developed countries is a consequence of the modern hygienic conditions and fewer bacterial, viral, and parasitic infections during infancy and childhood (26). Although fundamental to reduce infectious diseases, an excessively hygienic environment in early life might induce adverse effects on the host microbiome, altering certain strains of necessary commensal bacteria (dysbiosis). Furthermore, microbial dysbiosis might arise from the modern lifestyle that is characterized by limited physical activity, low intake of fibers, a diet high in saturated fats, and more frequent use of antibiotics. An impaired microbiota might also result from early life events such as cesarean section, premature birth, early antibiotic exposure, and formula feeding (Table 2) (27). Patients with EoE showed differences in the esophageal microbiome and an increase of bacterial load compared to patients with GERD and healthy controls (28, 29, 62). Harris et al. have demonstrated that the esophageal microbiome in children with untreated and active EoE is characterized by the predominance of *Haemophilus* strain, compared to patients with disease-remission and healthy controls (29). Also, Benitez et al. characterized the bacterial composition of the oral and esophageal microenvironments from children with EoE and healthy controls, showing that specific bacterial strains (mainly Firmicutes) were more abundant in the esophagus compared to the oral cavity in EoE patients (62). These data suggest that eosinophilic inflammation might specifically alter the esophageal microbiota, and the oral microbiota could not be used as a surrogate for monitoring the disease activity.

**Table 2:** Putative early risk factors of eosinophilic esophagitis (EoE).

Microbial gut dysbiosis	Microbial dysbiosis might arise from a modern lifestyle (limited physical activity, low intake of fibers, high saturated fats in the diet, and frequent use of antibiotics) and early life events (cesarean section, premature birth, early antibiotic exposure, and formula feeding) (25, 27–29)
Monogenic diseases	Hyper-IgE syndrome, Ehlers-Danlos syndrome, ERBIN deficiency, Loeys-Dietz syndrome, Netherton's syndrome, PTEN hamartoma tumor syndrome, severe atopy syndrome associated with metabolic wasting syndrome (7)
Esophageal atresia (EA)	EA and EoE might share same risk factors: genes, early life factors (prematurity, NICU admission), early exposure to acid suppressants and antibiotics, GERD and esophageal dysmotility and epithelial injury (30–33)
Esophageal injury in childhood, and fetal chest malformations	Caustic damage and diaphragmatic hernia might allow the development of EoE with mechanisms not well-understood and investigated (34, 35)
Western diet and obesity	A recent study in mice demonstrated that a high fat diet and obesity aggravated the immune histopathological characteristics and increased inflammatory cells in the EoE experimental model (36)
Low level of vitamin D	The supplementation of vitamin D *in utero* and early life seems to reduce the risk of atopy (37–43)
Early life exposures	Cesarean section, preterm birth, NICU admission, formula feeding, early prescription of PPI, and antibiotics might impair the host microbiome and the developing immature immune system (44–49)
Early prescription and long-term therapy with proton pump inhibitors (PPI)	PPIs prevent the digestion of food allergens, increase the gastric permeability, and alter the intestinal microbiome (27, 49–56)
Early prescription of antibiotics	Antibiotics might impair the immature gut microbiome, that is essential for the developing of immune system (27, 49, 57–59)
Formula feeding	Human milk shows potentially anti-allergic immune properties and is fundamental for the correct development of a well-balanced gut microbiome (27, 60, 61)

Evidence on the role of the microbiome in EoE pathogenesis is still limited to a few studies. However, two possible hypotheses could explain the relationship between the gut microbiome and EoE: (1) early life risk factors might specifically influence the correct development of the esophageal microbiome, predisposing to EoE, (2) eosinophilic inflammation could lead to esophageal dysmotility and decrease the esophageal compliance; thus EoE itself might induce esophageal microbiome alteration (28). Both hypotheses might coexist in a vicious circle, and the first one opens the unexplored field of the early prevention of EoE. Currently, only a single study in a murine model showed the beneficial effect of the probiotic *Lactococcus lactis* NCC 2287 on the esophageal inflammation (63). Although raising evidence explained the pivotal role of the well-balanced gut microbiome in the correct development of the immune system (25), the precise mechanisms whereby hygienic environment and dysbiosis interact with each other and result in allergic and autoimmune disease is still understood (64). Moreover, further studies are needed to clarify the role of dysbiosis in EoE pathogenesis and to identify possible preventive strategies.

Infectious diseases might act as promotive or protective factors for atopic diseases, including the EoE. Studies reported the development of EoE after herpes simplex virus (HSV) infection in immunocompetent adults and children. These data suggest that HSV esophagitis might predispose to EoE, impairing the esophageal barrier, and increasing the epithelial permeability (11, 12).

In Western countries, the overall prevalence of *Helicobacter pylori* infection was decreased in the last decades, probably contributing to the rise of allergic diseases (65). Experiments in murine models demonstrated that the *H. pylori* infection early in life was protective against asthma through the induction of regulatory T cells (T-regs) (66). Furthermore, epidemiological data showed that the *H. pylori* infection was negatively associate with EoE, demonstrating the potential protective role in EoE pathogenesis (67–70). The decrease of H. *pylori* infection in Western countries might also be a consequence of better hygienic conditions; furthermore, its possible protective role might explain the lower prevalence of EoE in developing countries, where the infection is usually acquired in childhood. On the other hand, Molina-Infante et al. recently published the results of a large prospective case-control study conducted in 23 centers, and showed that the prevalence of *H. pylori* infection was not different between EoE cases and controls (37 vs. 40%; *p* = 0.3; OR 0.97; 95% CI 0.73–1.30), neither in children (42 vs. 46%; *p* = 0.1) nor in adults (36 vs. 38%; *p* = 0.4) (71). Therefore, there are already insufficient and conflicting data to support the protective role of *H. pylori* infection, and several issues are still open.

### Diseases of Modern Life and Phenotypes of EoE

Recent advances in disease pathogenesis and prognosis have demonstrated that EoE could be classified in different phenotypes based on specific comorbidities. Epidemiological data demonstrated that EoE is so strongly associated with atopic comorbidities (asthma, allergic rhinitis, IgE-mediated food allergy, atopic dermatitis) (3, 9, 72) to follow allergic conditions in the atopic march, as a late manifestation (73). However, a significant number of EoE patients do not present allergic diseases, suggesting a possible non-atopic phenotype (2). Interestingly, several reports have suggested that EoE may be more frequently associated with some non-allergic disorders, including connective tissue disorders (74), autoimmune diseases (coeliac disease) (8), and contradictorily inflammatory bowel diseases (IBD) (8, 75–77), that are increased in the last decades, especially in Western countries (8). The pathogenetic mechanisms explaining the association between these non-atopic diseases and EoE are poorly understood and investigated. EoE and coeliac disease (CD) are two inflammatory diseases induced by food allergens. Although CD resulted more frequent in EoE patients than controls (5.6% of EoE, 0.9% of non-EoE, *P* < 0.0001) (8), Lucendo et al. did not find a common genetic basis between these two diseases (78). The frequency of the HLA DQ2 and DQ8 alleles predisposing to CD was not observed in adult EoE patients compared to controls (78). Also, type 1 diabetes, cystic fibrosis, adrenal insufficiency, autism, attention deficit hyperactivity disorder (ADHD) (8), and monogenic diseases (7) appear to be significantly associated with a non-atopic phenotype of EoE (2).

An increasing amount of evidence showed that children with esophageal atresia (EA) (30–32) or with diaphragmatic hernia (34) are at higher risk to develop EoE (33, 34, 79). Several risk factors have been associated with the development of EoE in children with EA, such as early life factors, early exposure to acid suppressants and antibiotics, GERD, esophageal dysmotility, and epithelial injury (79). Interestingly, Krishnan et al. demonstrated that children with EoE + EA share the same dysregulated genes (that encode for proteins involved in epithelial barrier functions and Th2 inflammation) compared to patients with EoE and without EA (33).

Although not widely demonstrated, another possible risk factor for EoE might be childhood exposure to caustic ingestion. Homan et al. reported a case of EoE development after caustic damage in a child with allergic comorbidity (35). The authors proposed two possible explanations for this association: (1) the caustic ingestion primarily triggered the eosinophilic inflammation of the esophagus or (2) after caustic damage the esophageal lesion might allow the trigger exposure (mainly food allergens) that might lead to EoE (35). Although fascinating, this report is characterized by some bias (child presented allergic diseases); however, further and extensive studies are required to confirm this data.

The diagnosis of gastroesophageal reflux disease (GERD) was also increased in the last two decades in Western countries (80), in parallel to allergic diseases, and, as a result of cow's milk allergy in the half of infants with refractory GER (81). Some authors reported that GERD might play a role in the pathogenesis of esophageal eosinophilia, more relevant in PPI-responsive cases (82). GERD, esophageal eosinophilia, and EoE are not mutually exclusive and may coexist in the same patient. However, there was no precise data about this association, and four mechanisms were proposed to explain it. (1) GERD causes esophageal eosinophilia in the absence of EoE, (2) GERD and EoE coexist but are unrelated, (3) EoE contributes to or causes GERD, (4) GERD contributes to or causes EoE (82). In patients with GERD, acid reflux alters the epithelial barrier of the esophagus, increasing the permeability and the passage of food allergens that might trigger EoE. Furthermore, acid reflux in GERD may induce the expression of inflammatory molecules and eosinophil chemoattractants (13, 83). On the other hand, eosinophilic inflammation produces different molecules (vasoactive intestinal peptide and interleukine-6) that might impair the esophageal peristalsis and delay the esophageal acid clearance (14). The subepithelial fibrosis, a delayed complication of EoE, might promote esophageal dysmotility (15). Further studies are needed to understand if this possible pathogenetic correlation might early predispose children with GERD to develop the EoE.

Interestingly, the 10–15% of children with EoE presented to the otolaryngologist before to be referred to the gastroenterologist (84), and the 33% of these patients required one or more otolaryngologic surgical interventions (20% bilateral myringotomy, 14% tonsillectomy, 18.5% adenoidectomy, 1.4% sinus irrigation, 3.3% bronchoscopy, and 1.4% laryngotracheoplasty), suggesting that EoE might overlap with otolaryngologic pathology (85).

### Western Diet and Lifestyle

Although foods are the primary triggers of EoE, there are limited data about the role of the Western diet in the contribution of the EoE pathogenesis. Higher levels of fatty acids characterize the Western diet and could be related to the increased risk of developing allergic diseases. In a recent study in mice, Silva et al. demonstrated that high-fat diet and obesity aggravated the immune histopathological characteristics and increased inflammatory cells in the EoE experimental model (36). These fascinating data provide new insights about obesity as a possible risk factor, impairing EoE symptoms; however, further prospective studies are needed.

No studies evaluated tobacco exposure in children and adolescents with EoE. Only a recent case-control study of adult patients showed that smoking was inversely associated with EoE compared to controls (86).

### Geographic Risk Factors and Vitamin D Levels

As previously reported and already described for other inflammatory gastrointestinal diseases, EoE prevails in specific geographic areas of the world. Prevalence rates of EoE were higher in Western regions of Europe, North America, and Australia than Asia and Africa (3). These geographic differences between Western countries (high prevalence) and Eastern countries (low prevalence) suggest that environmental factors might play a significant role in etiological mechanisms. The effects of people migration on the future development of EoE have not yet been investigated.

A few and conflicting studies evaluated the geographic distribution of EoE, based on the population density. An extensive US survey of Spergel et al. showed that EoE prevalence was higher in urban (0.58) and suburban (0.44) compared with rural settings (0.36, *P* < 0.0065) (87). Lee et al. demonstrated no significant difference in the incidence of EoE between people living in the rural area (50.9%) vs. patients from the urban ones (49.1%) (88). On the other hand, more recently, Jensen et al. found a strong inverse association between the population density and development of esophageal eosinophilia or EoE, demonstrating that EoE was more common in rural areas, in contrast with the hygienic hypothesis (89). A possible explanation of these results might be the geographic variation of specific environmental allergens.

Eosinophilic esophagitis prevails in cold climate zones, suggesting a possible association with specific aeroallergens (tree or grass pollens) and with low serum vitamin D levels (18). Increasingly significant evidence showed a link between vitamin D deficiency (maternal diet during pregnancy, early childhood diet, lack of exposure to sunlight) and risk of atopy, as described for asthma, allergic rhinitis, food allergy, and atopic dermatitis (37–39). This association is generally strongest in early life; in fact, interventional studies showed that the supplementation of vitamin D *in utero* and early life reduces the risk of recurrent wheeze and asthma (40–43). Although vitamin D enhances antimicrobial pathways, promotes peripheral immunological tolerance, and maintains mucosal barrier integrity, no studies have evaluated its possible preventive role in EoE development or its help in disease remission.

Climate zones might also affect the season of EoE diagnosis. Several single-center studies have evaluated the seasonality of symptoms and new diagnoses of EoE. In pediatric cohort studies, the seasonal exposure to aeroallergens increased the esophageal eosinophilic inflammation in children with EoE and allergic rhinitis (90, 91). However, the association between EoE relapse and season is still unclear, and available results were contradictory (16, 17, 92–97).

## Early Life Risk Factors of EoE: State of Art

Early life is a critical period during the immune system and microbiota mature, becoming susceptible to early environmental exposures. A well-balanced microbiome is fundamental for the correct development of the immune system (98–100), and numerous early life exposures, including prenatal (maternal diseases, mother diet, and lifestyle), intrapartum (cesarean section, maternal fever, and infections, prematurity), and postnatal factors (early antibiotic and acid suppressants use, formula feeding), might impair the gut microbiome, and predispose to allergic diseases (101–109). The association between early impaired microbiota and risk of atopy is widely described for asthma, allergic rhinitis and food allergy (44, 45, 110). A few studies postulated that early life exposures might also predispose to EoE in childhood (Table 2). However, few studies focalized on early life exposures and their effects on the future development of EoE (27, 46–49). The available studies reported that formula feeding (27, 60), neonatal intensive care (NICU) admission, prematurity (47, 49), maternal fever (47), antibiotic and acid suppressants use in infancy (27, 49), cesarean delivery (27, 47) were putative early risk factors of EoE. The antibiotic and proton pump inhibitor (PPI) use in infancy showed the most consistent evidence of a positive correlation with the future development of EoE.

### Effect of Early-Life Use of PPIs and Antibiotics

Although PPIs are used to treat GERD and esophageal eosinophilia, some studies paradoxically showed that the early PPI use might predispose to the development of autoimmune gastrointestinal diseases (celiac disease) (60), food allergies (13), and EoE (50). Physiologically, digestion of food proteins–and potential food allergens–begins into the stomach through pepsin proteinases, that are activated by the gastric acid *milieu*. PPI therapy might inactive proteinases and facilitate the digestive escape of food allergens, increasing the gastric pH. Also, PPI might increase the gastric mucosal permeability and the passage of allergens through the gastric mucosa, allowing their exposure to immune cells and the activation of atopic inflammation (51–53). Finally, PPI might alter the esophageal microbiota, and the modulation of immune response (54, 55). The risk to develop EoE after PPI therapy later in life has minimally been evaluated and could be higher after a long-term therapy (56, 111, 112). However, these data suggest that the immune system of infants might be more susceptible to PPI exposure, which might trigger the allergen-mediated inflammation of EoE. Since 1989 when the first PPI (Omeprazole) has been introduced into clinical practice, a worldwide escalation of PPI prescriptions was described at any age. Surprisingly, a pediatric study documented an 11-fold increase of new PPI prescriptions under 12 months of age in the last two decades (113).

The use of antibiotics in pregnancy is related to the treatment of several infections, such as bacterial vaginosis and urinary tract infections. Also, *intrapartum* and *peripartum* antibiotic prophylaxis are fundamental to decrease the risk of Group B *Streptococcus* infection in positive mothers and newborns. However, antibiotics might alter the immature gut microbiome of the newborn. Studies in rodents demonstrated that the administration of antibiotics in pregnancy decreased the microbiota diversity and permanently altered the immunity (57, 58). In newborns, the early administration of antibiotics resulted in decreased *Bifidobacterium* and increased enterococci strains (59). The worldwide increase of antibiotics prescriptions, especially in infancy, might partially explain the rise of allergic diseases. Observational studies demonstrated that the early life antibiotic administration was associated with asthma, atopic dermatitis, and allergic rhinitis (114–116). As previously mentioned, Jensen et al. founded a significant association with early antibiotic use and the development of EoE in children (27). Although an exact cause-effect mechanism cannot be deducted, these data suggest that the early exposure to antibiotics potentially might alter the immature microbiome and the developing immune system, allowing the risk of EoE (117).

The worldwide increase of PPI and antibiotic prescriptions in early life, associated with their possible pathogenetic role in allergic disease and EoE, suggests a conscious and rational use of these drugs, especially in childhood.

### Breastfeeding and Timing of Food Introduction in Children

Breastfeeding might be a possible factor that could prevent the development of food allergy through different mechanisms. Human milk shows potentially anti-allergic immune properties; in particular, the presence of maternal antibodies might prevent exposure to food allergens and induce oral immuno-tolerance (118). However, there is limited evidence on the direct correlation between breastfeeding and the development of EoE. In a pediatric case-control study, Jensen et al. identified a strong interaction between the calpain14 (CAPN14) gene variant (rs6736278) and breastfeeding, suggesting the possible protective role of human milk against EoE. CAPN14 is a cysteine protease and plays a fundamental role in the integrity of the esophageal epithelial barrier. Furthermore, its expression is only limited to the esophageal mucosa (119). CAPN14 expression was almost 4-fold increased in EoE patients compared to controls. Higher levels of CAPN14 expression are associated with the downregulation of desmoglein 1, filaggrin, and zonulin, which are pivotal proteins of the epithelial barrier (119). Although the exact mechanism of interaction between breastfeeding and CAPN14 is still unknown, human milk with its immunological properties might protect the esophagus from the epithelial barrier impairment and the development of EoE in patients with specific genotypes (120).

Over the last decade, food allergy research mainly focused on the timing of food introduction and oral tolerance. Murine models well-explained the concept of oral tolerance, and previous works showed how early and regular oral exposure to food allergens induced clinical tolerance and immunological changes. A large amount of evidence demonstrated that an early introduction of allergens might protect against the risk to develop IgE-mediated food allergy (61, 121, 122). In the last years, an increasing scientific interest focused on the diagnosis of non-IgE mediated food allergy, which often presents with a delayed onset of gastrointestinal symptoms. The EAT study evaluated data of non-IgE mediated symptoms (colic, vomiting, regurgitation, diarrhea, and constipation), demonstrating that infants in the early intervention arm reported significantly more non-IgE type symptoms than children in the standard intervention arm. However, rates of non-IgE mediated symptoms were equivalent in both groups at any time point, suggesting that the reporting of these symptoms did not depend on the introduction of the specific food allergen (121, 123). Further research is needed to understand if early food introduction could prevent non-IgE mediated food allergies, including EoE. Although the understanding of the EoE pathogenesis achieved notable progress, there are no published studies about the timing of food introduction in infancy and the future development of EoE.

### Genetic Risk Factors

EoE has a strong familiar hereditability pattern. Monozygotic twins had a 44% disease concordance, a 2-fold increase compared with dizygotic twins (5, 6). These data underly a complex interplay between genic loci and environmental exposures, through epigenetic mechanisms that are partially understood (6). Also, the relative risk to develop this disease in dizygotic twins might increase more than 10-fold compared to siblings. The increased rate of EoE development in dizygotic twins could be attributed to the same early-life environmental factors, previously mentioned.

The inheritance mechanism of EoE could be related to the effects of multiple single nucleotide gene polymorphisms (SNPs) that increase disease risk, depending on the environmental exposures and disease risk-modifying factors (119, 124). Several studies, including candidate-gene identification and genome-wide association studies (GWAS), have identified different genetic *loci* that are likely contributing to the development of EoE and mainly include thymic stromal lymphopoietin (TSLP), calpain 14 (CAPN14), EMSY, LRRC32, STAT6, and ANKRD27 (7). These genetic loci are mainly involved in T-helper 2 type inflammation (allergic inflammation) and epithelial barrier function and integrity. Interestingly, EoE is also associated with several monogenic inherited diseases, especially with connective tissue disorders and skin diseases. Connective tissue disorders, such as Marfan and Ehlers Danlos Syndromes, share a common pathogenic mechanism through the dysregulation of the TGF-β signaling. Children with autosomal dominant Hyper-IgE Syndrome (HIES) and Netherton Syndrome have also significantly increased the incidence of EoE (125, 126). Defects in PTEN, dehydrogenase E1, and transketolase domain–containing 1 (DHTKD1) genes are also associated with EoE (127, 128).

## How Could we Prevent EoE?

The rise of EoE diagnosis, especially in children, is an actual problem, and preventive strategies are needed to limit this phenomenon. Although there are no published studies about the prevention of EoE, we could speculate that possible strategies of primary prevention of EoE might be:

1. Sustaining breastfeeding in the first 6 months of life, especially in preterm babies and newborns from mothers that underwent cesarean section.
2. Limiting the uncontrolled prescriptions of acid suppressants and antibiotics only in specific and right circumstances.
3. Do not delay the introduction of food allergens in infants.
4. Providing adequate levels of vitamin D in infant and children, especially in those from cold climate regions.
5. Encouraging a well-balanced diet and a healthy lifestyle both in pregnant women both in children.

This work has several strengths. Firstly, this is a comprehensive review, summarizing the current knowledge on EoE risk factors, and focusing on the role of early exposures. Also, this review tried to answer to two main clinical issues: (1) the increased prevalence and incidence of EoE in Western countries, especially in children; (2) the lack of knowledge on early risk factors and possible preventive strategies.

There are several limitations. First of all, the lack of extensive and prospective studies evaluating the real burden of environmental risk factors, particularly the pathogenetic role of early exposures. Secondly, the vast majority of genetic and epidemiological studies were realized in Western Countries and mostly in the US. Finally, a few studies evaluated the gene-environmental interactions and the possible preventive strategies for EoE. Therefore, the lack of prospective and extensive studies from Eastern and developing Countries did not allow to draw reliable conclusions on the role of early risk factors and preventive strategies in EoE.

In conclusion, EoE is an emerging atopic disease that affects people at any age and characterized by symptoms due to esophageal inflammation, dysmotility, and fibrosis. As described for allergic diseases, several environmental risk factors and early-life exposures might interfere with genes, alter tolerance mechanisms, and activate the Th2 inflammation of EoE. Further studies are needed to identify risk factors of EoE, understand the interaction between genes and environment, finally find possible early preventive strategies.

## Author Contributions

MV and MD reviewed the literature and wrote the manuscript. AL, SC, IB, and GM reviewed the literature and helped with the writing of the manuscript. All authors approved the final version of the manuscript and agreed to be accountable for all aspects of the work. Questions related to the accuracy or integrity of any part of the work are appropriately investigated and resolved.

## Conflict of Interest

The authors declare that the research was conducted in the absence of any commercial or financial relationships that could be construed as a potential conflict of interest.

## Abbreviations

AA: arachidonic acid
ADHD: attention deficit hyperactivity disorder
CAPN14: calpain 14
DHA: docosahexaenoic acid
EA: esophageal atresia
EoE: eosinophilic esophagitis
EPA: eicosapentaenoic acid
GERD: gastroesophageal reflux disease
HSV: herpes simplex virus
NICU: neonatal intensive care unit
OIT: oral immunotherapy
PPI: proton pump inhibitor
PUFA: polinsatured fatty acid
SLIT: sublingual immunotherapy
Th2: T helper type 2
TLSP: thymic stromal lymphopoietin
T-regs: regulatory T cells.
